# Differences in the cerebral amyloid angiopathy proteome in Alzheimer’s disease and mild cognitive impairment

**DOI:** 10.1007/s00401-024-02767-1

**Published:** 2024-07-22

**Authors:** Dominique Leitner, Tomas Kavanagh, Evgeny Kanshin, Kaleah Balcomb, Geoffrey Pires, Manon Thierry, Jianina I. Suazo, Julie Schneider, Beatrix Ueberheide, Eleanor Drummond, Thomas Wisniewski

**Affiliations:** 1 Center for Cognitive Neurology, New York University Grossman School of Medicine, New York, NY 10016 USA; 2 Comprehensive Epilepsy Center, New York University Grossman School of Medicine, New York, NY 10016 USA; 3 Department of Neurology, New York University Grossman School of Medicine, New York, NY 10016 USA; 4 Brain and Mind Centre and School of Medical Sciences, University of Sydney, Camperdown, NSW 2050 Australia; 5 Proteomics Laboratory, Division of Advanced Research Technologies and Department of Biochemistry and Molecular Pharmacology, New York University Grossman School of Medicine, New York, NY 10016 USA; 6 Department of Biochemistry and Molecular Pharmacology, New York University Grossman School of Medicine, New York, NY 10016 USA; 7 Department Rush Alzheimer’s Disease Center, Rush University Medical Center, 1750 W Harrison Street, Suite 1000, Chicago, IL 60612 USA; 8 Department of Neurological Sciences, Rush University Medical Center, Chicago, IL USA; 9 Department of Pathology, Rush University Medical Center, Chicago, IL USA; 10 Department of Pathology, New York University Grossman School of Medicine, New York, NY 10016 USA; 11 Department of Psychiatry, New York University Grossman School of Medicine, New York, NY 10016 USA

**Keywords:** Cerebral amyloid angiopathy, Proteomics, Alzheimer’s disease

## Abstract

Cerebral amyloid angiopathy (CAA) is characterized by amyloid beta (Aβ) deposition in cerebrovasculature. It is prevalent with aging and Alzheimer’s disease (AD), associated with intracerebral hemorrhage, and contributes to cognitive deficits. To better understand molecular mechanisms, CAA(+) and CAA(−) vessels were microdissected from paraffin-embedded autopsy temporal cortex of age-matched Control (*n *= 10), mild cognitive impairment (MCI; *n *= 4), and sporadic AD (*n *= 6) cases, followed by label-free quantitative mass spectrometry. 257 proteins were differentially abundant in CAA(+) vessels compared to neighboring CAA(−) vessels in MCI, and 289 in AD (*p *< 0.05, fold-change > 1.5). 84 proteins changed in the same direction in both groups, and many changed in the same direction among proteins significant in at least one group (*p *< 0.0001, *R*^2^ = 0.62). In CAA(+) vessels, proteins significantly increased in both AD and MCI were particularly associated with collagen-containing extracellular matrix, while proteins associated with ribonucleoprotein complex were significantly decreased in both AD and MCI. In neighboring CAA(−) vessels, 61 proteins were differentially abundant in MCI, and 112 in AD when compared to Control cases. Increased proteins in CAA(−) vessels were associated with extracellular matrix, external encapsulating structure, and collagen-containing extracellular matrix in MCI; collagen trimer in AD. Twenty two proteins were increased in CAA(−) vessels of both AD and MCI. Comparison of the CAA proteome with published amyloid-plaque proteomic datasets identified many proteins similarly enriched in CAA and plaques, as well as a protein subset hypothesized as preferentially enriched in CAA when compared to plaques. SEMA3G emerged as a CAA specific marker, validated immunohistochemically and with correlation to pathology levels (*p *< 0.0001; *R*^2^ = 0.90). Overall, the CAA(−) vessel proteomes indicated changes in vessel integrity in AD and MCI in the absence of Aβ, and the CAA(+) vessel proteome was similar in MCI and AD, which was associated with vascular matrix reorganization, protein translation deficits, and blood brain barrier breakdown.

**Supplementary Information:**

The online version contains supplementary material available at 10.1007/s00401-024-02767-1.

## Introduction

Cerebral amyloid angiopathy (CAA) is characterized by amyloid beta (Aβ) deposition in the cerebrovasculature. It is not only prevalent in aging and in almost all Alzheimer’s disease (AD) patients but can also occur on its own, independent of other AD-related pathology [6, 18, 30, 70]. The presence and severity of CAA promotes the progression of AD-related clinical symptoms, and is also associated with more rapid cognitive decline in normal aged subjects [3, 4, 6]. The development of CAA may contribute to cognitive decline directly by facilitating hypoxia and neuronal injury or indirectly by promoting tau pathology [41, 52, 70]. CAA is linked to cerebral hemorrhages and amyloid-related brain imaging (ARIA) abnormalities, a major complication of AD immunotherapeutics [6, 19, 22, 47, 48, 50, 57, 61, 68].

There are some mechanistic components of CAA development that have been elucidated, but the underlying pathogenesis is not well understood. Aβ deposition in CAA usually occurs in a spiral-like fashion, distributed in a characteristic patchy pattern, often in an adventitial manner rather than medially in larger arterioles, and most commonly consists of the more soluble Aβ40 over less soluble Aβ42 [6]. It has been proposed that Aβ expands from the basement membrane and replaces elements of the vessel wall with disease progression, potentially impacting neighboring cells including neurons, inflammatory cells, endothelial cells, and the integrity of the blood brain barrier (BBB) [6]. Increased BBB permeability has been observed in CAA patients and animal models, along with cerebral hypoperfusion that occurs before clinical dementia [15, 40, 58]. Previous proteomic studies aiming to identify CAA-associated proteins have largely relied on blood vessel extraction using density gradient approaches, which results in a mixture of CAA(+) and CAA(−) vessels, making it difficult to parse proteins selectively enriched in CAA(+) vessels [45, 72, 75]. An alternative approach has been to identify CAA-associated proteins in large tissue regions enriched in CAA [23]. Only two studies to date have used laser capture microdissection (LCM) to selectively capture CAA(+) vessels from AD tissue for proteomic analyses [20, 26]. These previous studies have identified multiple CAA-associated proteins such as HTRA1, SMOC1, SMOC2, and SRPX1 [20, 23, 26, 45, 72, 75]. However it is still not well understood which proteins are selectively enriched in CAA(+) vessels in comparison to neighboring CAA(−) vessels, whether the CAA proteome is the same in early and advanced AD, and whether the same proteins are enriched in CAA and amyloid plaques.

In this study we leveraged our localized proteomic approach to allow for distinct CAA(+) and CAA(−) vessel proteome characterization. The aims of our study were to: (1) characterize the proteins selectively enriched in CAA(+) vessels, (2) compare the protein composition of CAA(+) vessels in early AD (mild cognitive impairment [MCI]) versus advanced AD, (3) characterize the proteome of neighboring CAA(−) vessels in MCI and AD, (4) compare the protein composition of CAA(−) vessels in MCI and AD, and (5) compare the CAA(+) vessel proteome to the amyloid-plaque proteome.

## Material and methods

### Brain tissue

Brain tissue was acquired under protocols with Institutional Review Board (IRB) approval at NYU Grossman School of Medicine and Rush University. Cases are a part of the Religious Orders Study (ROS) and Memory and Aging Project (MAP) cohorts [2]. Clinical assessment and neuropathology was performed at Rush University [4, 38, 54]. Cases were stratified into Control, MCI, and advanced AD experimental groups using a combination of both clinical and neuropathological criteria. Cases were initially stratified by the clinical cognitive final consensus diagnosis that was generated by a neurologist with expertise in dementia by a review of all available cognitive data that was blinded to post-mortem data. Cases were excluded if they were designated to have a clinical diagnosis of AD and another cause of cognitive impairment. The following neuropathological inclusion criteria was then used to refine case selection in each group: neuropathological ABC score of A0-1/B0-2/C0-1 for Control, A2-3/B1-2/C2-3 for MCI, and A3/B3/C3 for AD. Cases were prioritized to exclude those with high TDP-43 and Lewy body pathology. Formalin-fixed paraffin-embedded (FFPE) sections of small samples from the inferior temporal cortex were then obtained from Control (*n *= 62), MCI (*n *= 74), and AD cases (*n *= 56), which were then neuropathologically assessed to identify cases with sufficient CAA(+) vessels for localized proteomics analysis. Of these cases, *n *= 4 MCI and *n *= 6 AD had sufficient CAA(+) vessel area (2 mm^2^) for dissection in the inferior temporal cortex and were therefore selected for analysis. Control cases (*n *= 10) were included for comparison to CAA(−) vessels, and were age-matched to the other groups (*p *= 0.11, one-way ANOVA). Sample size was considered sufficient as in prior studies [10, 13, 25, 32, 64]. Case history is summarized in Table 1 and detailed further in Supplementary Table 1.

**Table 1:** Case History

Study group	Age	Sex	PMI (hours)	CAA score—temporal cortex (0–5)	ABC Score	*APOE* Genotype
Control	85.8 ± 6.4	7F/3M	8.9 ± 7.9	0.2 ± 0.4		
C1	82	M	7	1	A1, B1, C0	33
C2	91	F	15	0	A0, B2, C0	23
C3	80	M	17	0	A0, B1, C0	33
C4	81	F	5	0	A1, B1, C1	33
C5	93	F	3	0	A0, B2, C0	23
C6	90	F	26	0	A0, B1, C0	34
C7	87	M	4	0	A0, B1, C0	23
C8	76	F	6	0	A1, B0, C0	33
C9	83	F	1	0	A1, B1, C0	33
C10	95	F	5	1	A1, B1, C0	33
MCI	93.5 ± 6.7	3F/1M	7.9 ± 3.8	3.5 ± 1.9		
M1	84	F	6	5	A2, B2, C1	44
M2	94	F	12	5	A2, B1, C2	33
M3	95	F	N/A	3	A2, B2, C2	34
M4	101	M	6	1	A3, B2, C2	23
AD	86.4 ± 4.9	5F/1M	10.8 ± 8.9	5.0 ± 0.0		
AD1	88	F	26	5	A3, B3, C3	33
AD2	81	F	6	5	A3, B3, C3	34
AD3	93	M	6	5	A3, B3, C3	34
AD4	83	F	2	5	A3, B3, C3	44
AD5	84	F	17	5	A3, B3, C3	44
AD6	90	F	8	5	A3, B3, C3	34

Mean and standard deviation are indicated for group values

*N/A* not available

### Screening immunohistochemistry (IHC)

To identify cases with sufficient CAA(+) vessels for localized proteomics studies, one standard glass slide per case was sectioned from FFPE tissue by the NYU Center for Biospecimen Research and Development Core. Briefly, 8 µm sections were deparaffinized and rehydrated through a series of xylenes and ethanol washes, followed by antigen retrieval with 88% formic acid, and 10 mM sodium citrate with 0.05% tween at pH 6. Sections were blocked in 10% normal horse serum for 1 h, and incubated in a combination of 4G8 (1:1000, BioLegend #800711) and 6E10 (1:1000, BioLegend #803017) primary antibodies targeting Aβ overnight at 4 °C. After washing, sections were incubated in biotinylated mouse secondary antibody (1:1000, Vector Laboratories) for 1 h, followed by avidin–biotin peroxidase (Vector Laboratories) for 1 h. After washing, sections were incubated with diaminobenzidine (DAB) chromogen solution (ThermoFisher). After additional washes, sections were coverslipped. A regional CAA score (0–5) and semiquantitative frequency of intravascular and perivascular score (+, ++, +++) was given for each slide to indicate relative level of pathology, excluding leptomeninges. Cases were included with intravascular and/or perivascular scores of +++ for MCI and AD cases, as well as microscopic confirmation of adequate CAA(+) vessels present.

### Laser capture microdissection (LCM)

FFPE tissue was cut into 8 µm sections onto LCM PET membrane slides [12, 34, 35, 51] by the NYU Experimental Pathology Core. CAA(+) (Aβ positive) vessels and CAA(−) (Aβ negative; non-CAA) vessels were visualized by IHC with 4G8 antibody followed by DAB chromogen counterstained with hematoxylin for all cases. Briefly, sections were deparaffinized and rehydrated through a series of xylenes and ethanol washes, followed by incubation in 0.3% H_2_O_2_ for 20 min, blocking in 10% normal goat serum for 1 h, and incubated with 4G8 primary antibody (1:1000, BioLegend #800711) overnight at 4 °C. After washing, sections were incubated in biotinylated mouse secondary antibody (1:1000, Vector Laboratories) for 1 h, followed by avidin–biotin peroxidase (Vector Laboratories) for 1 h. After washing, sections were incubated with DAB solution (ThermoFisher) for 10 min. Finally, sections were counterstained with hematoxylin (Sigma #MHS16) and air dried overnight in a loosely closed container.

**Fig. 1:**
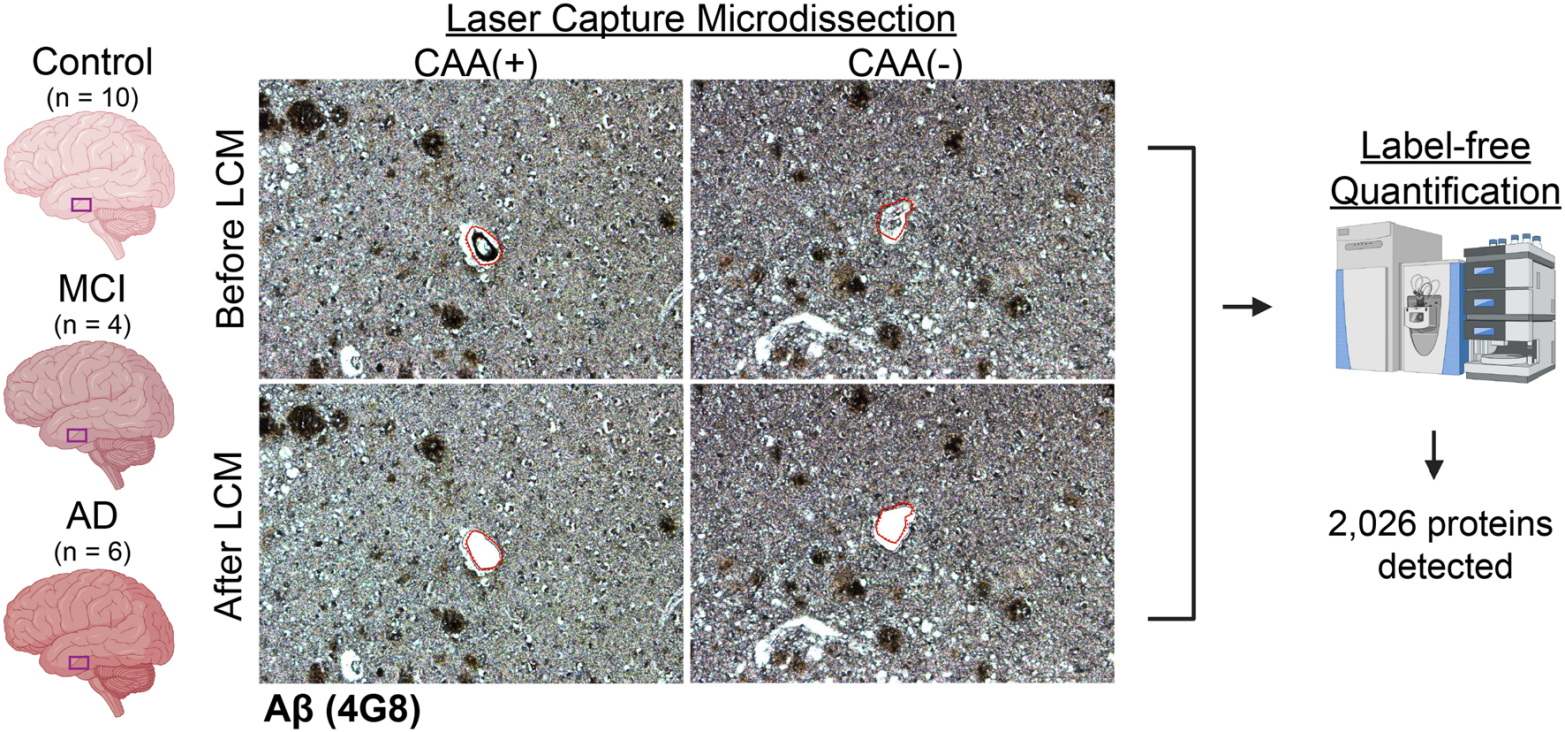
Schematic approach overview for LCM of CAA(+) and CAA(−) vessels. Blood vessels (2 mm^2^) from the inferior temporal cortex were microdissected by LCM from FFPE autopsy brain tissue from control (*n *= 10), MCI (*n *= 4), and AD (*n *= 6) cases. Both CAA(+) (Aβ positive) and neighboring CAA(−) (Aβ negative) vessels were dissected separately, with only CAA(−) vessels from Control cases. Proteins were quantified by label-free quantitative mass spectrometry to identify protein differences. There were 2026 proteins detected in at least half of one group

Blood vessels with a lumen and vessel wall in gray matter were microdissected at a consistent area per case of 2 mm^2^ into mass spectrometry (MS) grade water (Thermo Scientific) from *n *= 10 Control [CAA(−) vessels], *n *= 4 MCI [CAA(+) vessels and CAA(−) vessels collected separately], and *n *= 6 AD [CAA(+) vessels and CAA(−) vessels collected separately] cases. Both larger vessels and capillaries were included. Vessels in the leptomeninges and white matter were excluded. Microdissected samples were centrifuged for 2 min at 14,000* g* and stored at − 80 °C. LCM was performed at 10× magnification with a Leica LMD6500 microscope equipped with a UV laser. Schematic overview depicts workflow, partially generated in Biorender (Fig. 1).

### Label-free quantitative mass spectrometry (LFQ-MS)

Tissue samples were solubilized and digested using the modified SPEED sample prep workflow [9] by the NYU Proteomics Laboratory. Proteins were extracted in 10 µl of formic acid (FA) for 5 min at 73 °C. FA was then neutralized with 90 µl of 2 M TRIS containing 10 mM TCEP and 20 mM chloroacetamide (CAA), and samples were incubated at 90 °C for 30 min. For enzymatic digestion, samples were diluted 6× (v:v) with water containing 0.2 µg of sequencing-grade trypsin (Promega) and digested at 37 °C overnight. Digestion was halted with acidification to 2% of trifluoroacetic acid (TFA). Peptides were loaded on Evosep Pure C18 tips and separated on Evosep One LC system using 88 min ACN gradient (SPD15 method) on the analytical column packed with Dr Maisch C18 AQ 1.9 µm C18 beads (150 µm ID, 15 cm length, #EV-1106). Peptides were identified and quantified in data-independent (DIA) acquisition mode on a QExactive HF-X mass spectrometer (ThermoFisher). High-resolution full MS spectra were acquired with a resolution of 120,000, an AGC target of 3e6, a maximum ion injection time of 60 ms, and a scan range of 350–1650 *m*/*z*. Following each full MS scan, 22 data-independent HCD MS/MS scans were acquired at the resolution of 30,000, AGC target of 3e6, and stepped NCE of 22.5, 25, and 27.5.

MS raw data were analyzed using Spectronaut software (https://biognosys.com/shop/spectronaut) in directDIA (library-free) pipeline against the *Homo sapiens* UniProt reference database (http://www.uniprot.org/) concatenated with a list of common lab contaminants. Database searches were performed in the integrated search engine Pulsar. Independent quantification of Aβ was manually curated and included in search results, as in previous studies [10, 55]. Aβ intensity was determined by integrating the area under the curve for peptide LVFFAEDVGSNK (Aβ peptide). This peptide corresponds to amino acids 17–28 of Aβ, does not discriminate from cleaved or full length sequences, but this peptide does show strong enrichment and correlation to Aβ pathology [10, 20, 31, 55]. Data were log transformed and normalized using median intensity across all samples. Subsequent data analysis was performed in Perseus [67] (http://www.perseus-framework.org/), the R environment (http://www.r-project.org/), or GraphPad Prism. Raw data is available on the MassIVE server (https://massive.ucsd.edu/) under accession MSV000094400.

The protein expression matrix (*n *= 2188) was filtered to contain only proteins that were human, non-lab contaminant, and quantified in at least 50% of samples from at least one of the five groups (*n *= 2026). For principal component analysis (PCA), missing values were imputed from the normal distribution with a width of 0.3 and a downshift of 1.8 (relative to measured protein intensity distribution) in Perseus [67]. Multiple linear regression was performed to determine whether sample type or disease group contributed to differences in PCA1, in GraphPad Prism. Paired *t* tests were performed in Perseus v. 1.6.2.3 [67] to detect significant changes in protein expression between CAA(+) and CAA(−) vessel samples in MCI and in AD. Unpaired *t* tests were performed in Perseus to detect significant changes in protein expression between CAA(−) vessel samples from Control cases compared to MCI or AD CAA(−) vessel samples. Significance was considered at *p *< 0.05 and fold-change > 1.5. Cell-type annotations were derived from a previous vascular single cell RNAseq study [71], which included an association of 13 cell-type annotations. Twelve cell-type annotations were detected in the current dataset, red blood cell proteins were not detected in the current study and duplicate gene IDs were excluded from annotations (303 annotations overlap with detected proteins). Cell-type enrichment analysis was performed among significantly altered proteins with a Fisher’s exact test in GraphPad Prism and the R environment. A comparison of proteins by disease group were evaluated by Venn diagram generated from InteractiVenn [21]. Correlation analyses were performed by Pearson correlation in GraphPad Prism. Unsupervised hierarchical clustering and heatmaps were evaluated in the R environment with *ComplexHeatmap*. Individual protein plots depict significance for pairwise comparisons performed.

### Gene ontology (GO) term enrichment analysis

GO cell component (GOCC) term over-representation analysis was performed in R v4.3.0 using the packages *enrichplot* v1.20.0, *clusterProfiler* 4.8.1, with the genome wide annotation for human, *org.Hs.eg.db* v3.17.0. GO over-representation analysis was performed on gene subsets, with increased and decreased proteins separated, in CAA(+) vessels in MCI or AD and for proteins altered in CAA(−) vessels of MCI vs Control and AD vs Control. Prior to analyses, gene IDs were mapped to Entrez IDs with the ‘bitr’ function of *clusterProfiler*. GO terms were assessed for enrichment via one-way Fisher’s exact test, and multiple comparisons correction was performed with Benjamini–Hochberg to obtain an adjusted *p* value. GO terms were filtered to include those at an adjusted *p *< 0.05. The full list of proteins detected across all samples was used as the background list (2026 proteins) as this is a highly targeted dataset. ‘Lollypop’ plots of the top 10 up and down annotations were generated in R with the packages *ggplot2* v3.4.2 and *ggpubr* v0.6.0. *Simplify* function at 0.7 semantic similarity was used to reduce redundant terms in figures.

### Comparison with previous studies

Proteins that were significantly enriched or depleted in amyloid plaques in comparison to neighboring non-plaque tissue were sourced from published datasets that identified proteins significantly enriched/depleted in amyloid plaques in early onset AD and Down syndrome [10], and proteins significantly enriched/depleted in amyloid plaques in sporadic AD and preclinical AD [73]. Proteins were considered significantly enriched or depleted in plaques at *p *< 0.05 (*t *test) and fold change > 1.5 between plaque and non-plaque tissue for [10] and a ratio > 1.5 between plaque and non-plaque tissue for [73]. Remaining proteins that were detected, but not significant between groups were designated “present”. For this comparative analysis, proteins were designated as significantly enriched or depleted in CAA(+) vessels from proteins that were either significantly different between CAA(+) and CAA(−) vessels or exclusive identification in the majority of either CAA(+) or CAA(−) vessels within a disease group (i.e. ≥ 3 samples for MCI or ≥ 4 samples for AD) and 0 samples in the comparative blood vessel group. Remaining proteins that were detected, but not significant between groups were designated “present”. Proteins that were enriched in at least one CAA(+) vessel analysis and at least one plaque analysis were considered to be “CAA and plaque enriched” proteins. Proteins that were enriched in at least one CAA(+) vessel analysis and present or depleted in at least one plaque analysis and vice versa were considered to be “CAA enriched” or “plaque enriched” proteins, respectively. Proteins enriched only in one type of pathology and not detected in the other type of pathology were considered potentially enriched when found in more than half of the pathology groups (i.e., both CAA groups or 3 of 4 plaque groups). Proteins that were inconsistently enriched or depleted within CAA(+) vessel experimental groups or within plaque experimental groups were excluded from the analysis.

### Validation IHC

Follow up histology was performed on COL6A2, SEMA3G, and SMOC1 in inferior temporal cortex sections from progressive stages of disease [Control (*n *= 12), Preclinical AD (*n *= 10), MCI (*n *= 12), AD (*n *= 12)]. Cases were age-matched between groups (*p *= 0.35, one-way ANOVA), and included all cases evaluated by proteomics as well as additional cases to increase the power of the analysis. Additional AD and MCI cases were selected from those with the highest CAA and plaque scores by IHC screening studies. All Control cases that had a combined plaque and CAA score of ≥ 3 in IHC screening studies (indicating presence of moderate amyloid pathology in the absence of cognitive impairment) were included in the preclinical AD group. Additional Control cases were selected from cases that had a score of 0 for both plaques and CAA in IHC screening studies. Where possible, groups were balanced for sex, age, and *APOE* genotype, detailed in Supplementary Table 1. Briefly, FFPE sections (8 µm) were deparaffinized and rehydrated in a series of xylenes and ethanol dilutions. After washing, sections were incubated in 88% formic acid for 7 min for COL6A2 and SEMA3G, and 99% formic acid for 7 min for SMOC1. Heat-induced antigen retrieval was performed with 10 mM sodium citrate, 0.05% triton-x 100 pH 6 for COL6A2 and SEMA3G; and 10 mM sodium citrate, 0.05% Tween-20 pH 6 for SMOC1. Blocking with 10% normal goat serum or normal donkey serum was followed by primary antibodies COL6A2 (1:100, Novus Biologicals #H00001292-M01), Aβ40 (1:100, provided by Pankaj Mehta [44]), SEMA3G (1:100, Sigma HPA001761), SMOC1 (1:100, Abcam ab200219), and 4G8 (1:1000, BioLegend #800701) overnight at 4 °C. Sections were incubated with goat anti-mouse 647 and goat anti-rabbit 488 secondary antibodies (1:500, Jackson ImmunoResearch Laboratories), donkey anti-rabbit 647 and donkey anti-mouse 488 (1:1000, ThermoFisher), counterstained with DAPI (Sigma D9542) or Hoescht 33342 (1:1000, Sigma B2261), and coverslipped. Whole slide images were acquired using a Leica Aperio Versa 8 Scanner or an Olympus VS200 Slide Scanner at 10× magnification, with empty channel 568 used to capture autofluorescence for subtraction. For COL6A2 and SEMA3G, representative 40× images were obtained with the Leica Aperio Versa 8 Scanner, and for SMOC1, representative 60× images were captured on a Nikon C2 Confocal microscope.

### IHC quantification

Slide scan images for COL6A2, SEMA3G, and SMOC1 were imported into QuPath (v0.5.0) and gray matter manually annotated to exclude tissue folds, tears, and staining artifacts. A blood vessel classifier was trained using the autofluorescence channel (568) and applied to gray matter annotations in all images. Each image was subsequently checked to ensure classifier accuracy. Images were exported as .tif tiles with corresponding masks for blood vessels and total gray matter. Autofluorescence was subtracted from Aβ and COL6A2, SEMA3G, or SMOC1 channels in ImageJ2 (v2.14.0/1.54f). To determine COL6A2, SEMA3G, or SMOC1 positive staining in CAA(+) and CAA(−) vessels, blood vessel masks were applied to each image and areas of Aβ-positive or Aβ-negative staining subtracted, respectively. COL6A2, SEMA3G, or SMOC1 signal was then thresholded, and positive area and integrated density measured. For analysis, COL6A2, SEMA3G, or SMOC1 positive area in CAA(−) and CAA(+) vessels was normalized to total blood vessel area for each section. Integrated density was normalized to either CAA(−) or CAA(+) area to give average gray value per pixel. Results were visualized in GraphPad Prism (v10.0.3) and analyzed using Kruskal–Wallis test with Dunn’s post hoc test for the same pairwise comparisons performed in the proteomics analyses.

## Results

**Fig. 2:**
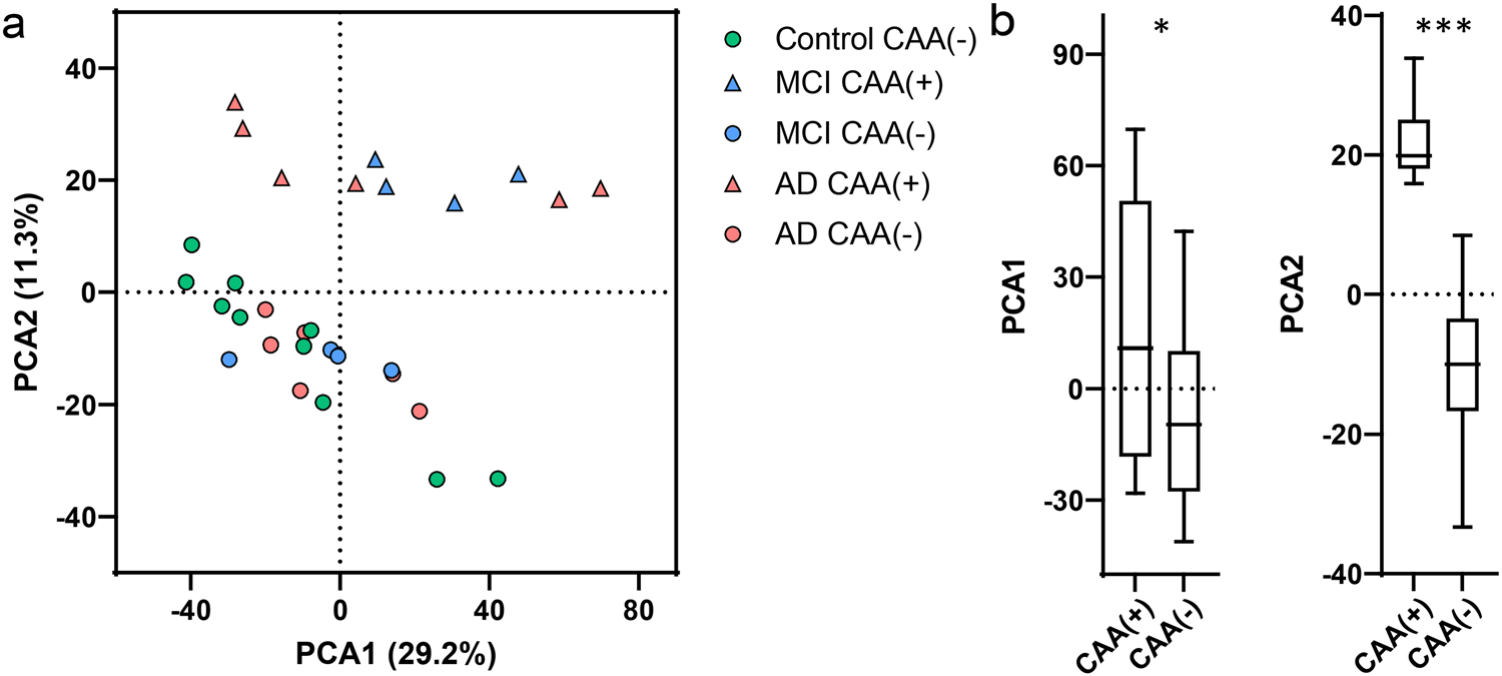
PCA of CAA(+) and CAA(−) vessels. **a** PCA shows distribution of Control, MCI, and AD CAA(+) and CAA(−) vessel samples. **b** There was significant segregation of CAA(+) vessel samples from CAA(−) vessel samples, particularly in PCA2 (*p *< 0.0001, unpaired *t* test), and some segregation was seen in PCA1 (*p *= 0.027). There was no segregation by disease group

From LFQ-MS, 2,026 proteins were detected in microdissected CAA(+) and CAA(−) blood vessels from age-matched Control (*n *= 10), MCI (*n *= 4), and AD (*n *= 6) cases (Fig. 1). PCA showed significant segregation of CAA(+) vessel samples from CAA(−) vessel samples in PCA2 (*p *< 0.0001), as well as some segregation in PCA1 (*p *= 0.027, Fig. 2). There was no segregation by disease group among all samples on the PCA nor was this seen by multiple linear regression (Supplementary Table 2).

**Fig. 3:**
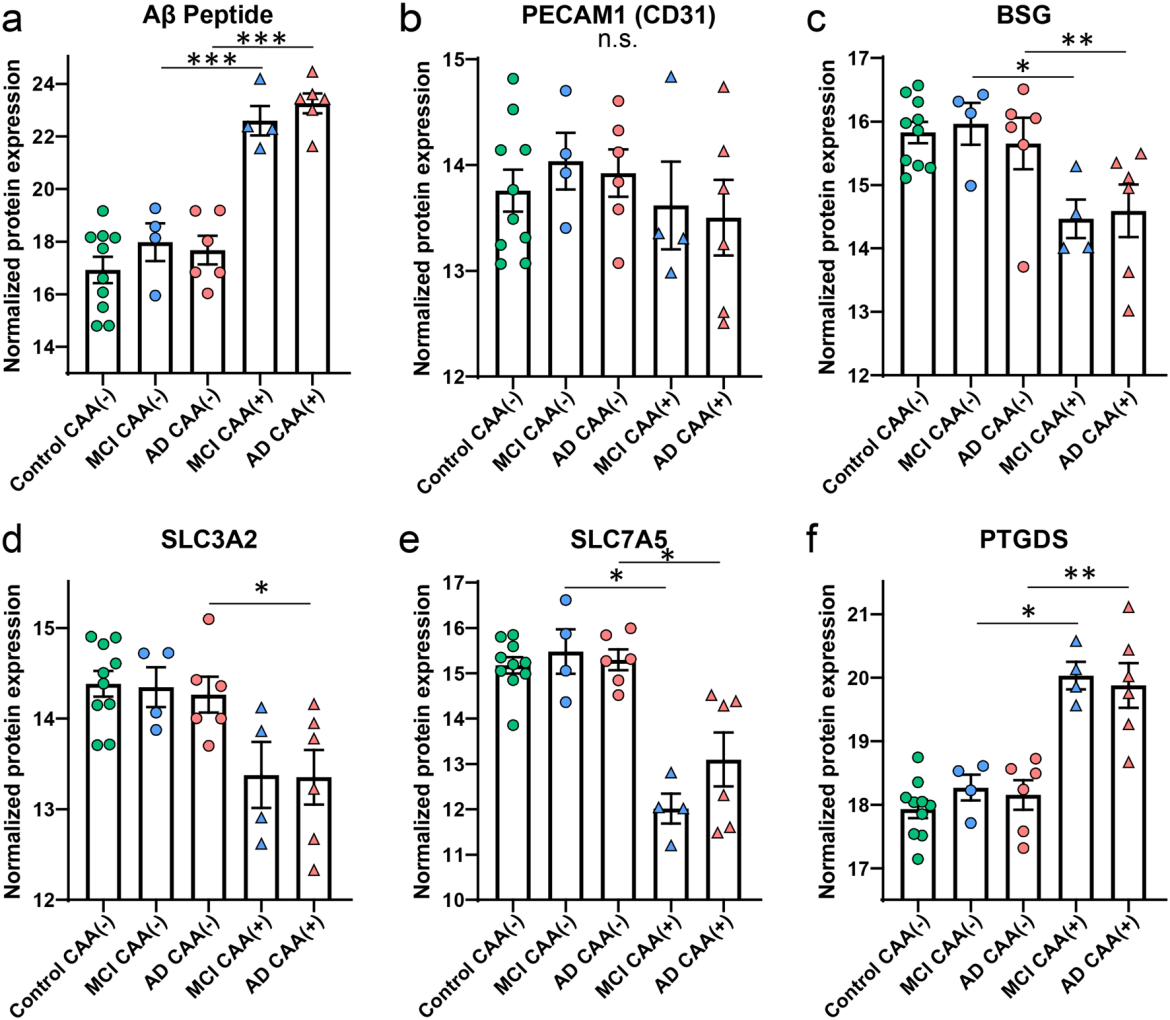
Markers of interest in dissected vessels. **a** The Aβ peptide was enriched in the CAA(+) vessels from both MCI and AD cases. **b** The endothelial cell marker PECAM1 (CD31) was detected in all samples and was not significantly different. **c**–**e** Evaluating specific vessel type markers, among those detected, capillary markers BSG, SLC7A5, and SLC3A2 were decreased in CAA(+) vessels. **f** A marker for veins, PTGDS, was increased in CAA(+) vessels. Significant pairwise comparisons are indicated for those analyses that were performed, *p < 0.05, **p < 0.01, ***p < 0.0001

Markers of interest were reviewed to characterize dissected samples, including the Aβ peptide and vessel markers. The Aβ peptide LVFFAEDVGSNK corresponds to amino acids 17–28 of Aβ, does not discriminate from cleaved or full length sequences, but this peptide does show strong enrichment and correlation to Aβ pathology [10, 20, 31, 55]. The Aβ peptide was significantly enriched in CAA(+) vessels in comparison to CAA(−) vessels in both MCI and AD (MCI: *p *= 7.80 × 10^–3^, 24.5-fold; AD: *p *= 2.07 × 10^–4^, 47.8-fold; Fig. 3a). The endothelial cell marker PECAM1 (CD31) [36] was detected in all samples and was present at a similar level in all groups (Fig. 3b). Specific blood vessel markers associated with capillaries or larger vessels [66] were detected (PTGDS, BSG, SLC3A2, SLC7A5, HSPA1A, PRSS23, TFRC). Among the detected markers, 4 were differentially abundant in AD CAA(+) vessels (PTGDS, BSG, SLC3A2, SLC7A5; Fig. 3c–f) and the same was observed in MCI except with no change in SLC3A2. Three of these proteins are associated with capillaries (BSG, SLC3A2, SLC7A5) and one with veins (PTGDS) [66]. In the CAA(−) vessels, these proteins were not altered in either disease group when compared to Control cases. These differences may indicate fewer CAA(+) capillaries in the microdissected CAA(+) vessels and a mix of vessel types in the CAA(−) vessels, consistent with what was observed when dissection was performed and with pathogenesis typically involving larger vessels [6].

### CAA(+) vs CAA(−) vessel comparisons

**Fig. 4:**
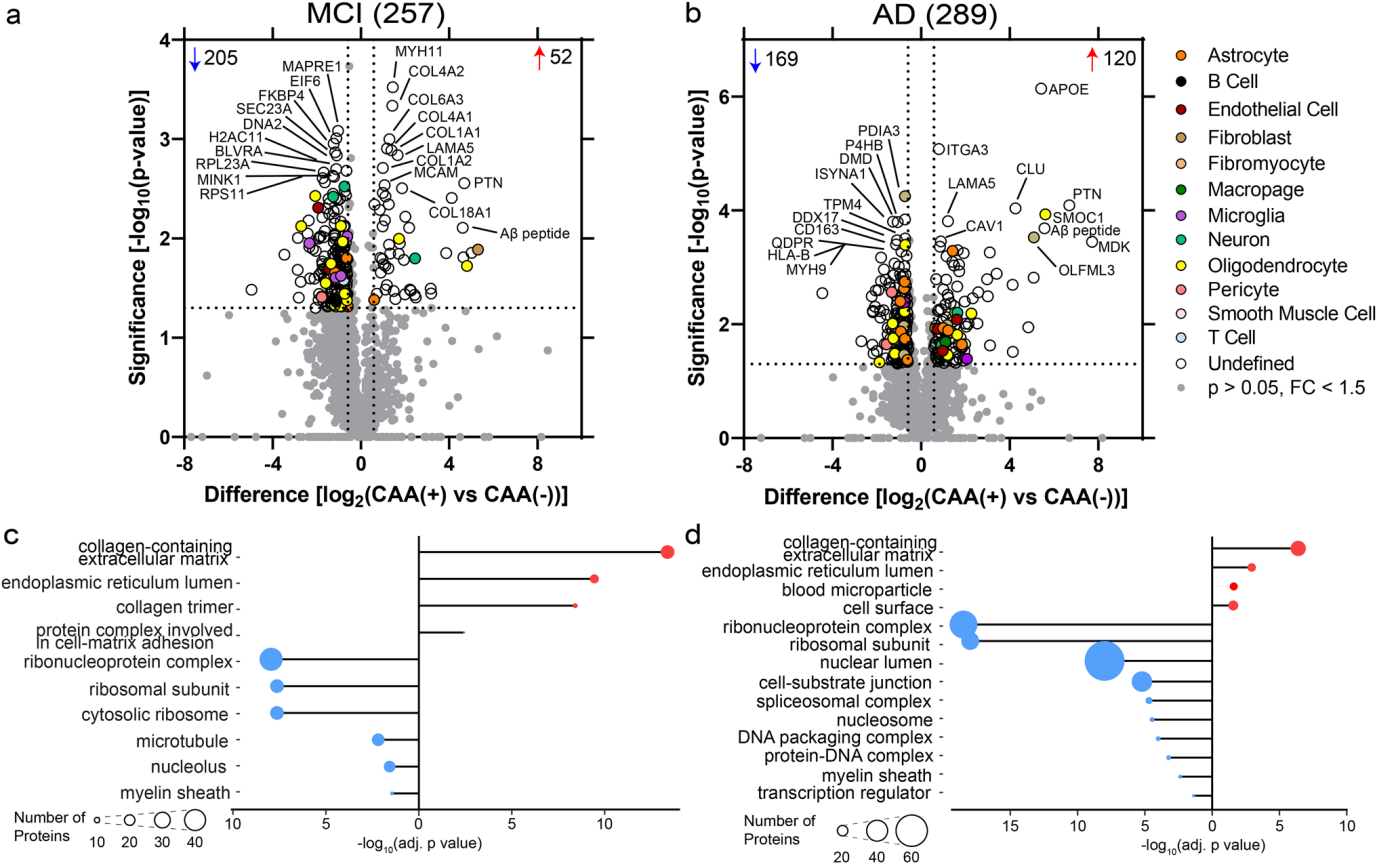
CAA(+) vs CAA(−) vessel differential expression analysis and GO cell component terms in MCI and AD. **a** When comparing MCI CAA(+) vessels to MCI CAA(−) vessels, there were 257 proteins differentially abundant (*p *< 0.05, fold change > 1.5), with 52 increased (red) and 205 decreased (blue). The top 10 significantly increased and decreased are annotated by gene name, as well as Aβ peptide when not among the top 10. **b** When comparing AD CAA(+) vessels to AD CAA(−) vessels, there were 289 proteins differentially abundant, with 120 increased and 169 decreased. Blood vessel specific cell-type annotations are indicated for each protein. **c** and **d** Differentially abundant proteins were associated with the indicated increased (red) or decreased (blue) GO cell component (GOCC) terms at adj. *p *< 0.05 in MCI and AD. Number of proteins associated with a term are depicted by circle size

Proteins differentially abundant in CAA(+) vessels when compared to neighboring CAA(−) vessels in each disease group were identified by paired *t* tests at *p *< 0.05 and with fold change > 1.5. There were 257 proteins differentially abundant in MCI, and 289 proteins in AD (Fig. 4a and b).

Cell-type annotations [71] were evaluated to determine cell-type enrichment of altered proteins. The majority of proteins were “Undefined” as they are expressed by multiple cell types or it is unknown, and thus would require other detailed follow up cell-type characterization analysis. From annotated proteins in MCI CAA(+) vessels, there was enrichment for astrocyte (*p *= 2.71 × 10^–2^) and oligodendrocyte (*p *= 5.70 × 10^–3^) proteins. The majority of the astrocyte proteins (75%; 3/4) and oligodendrocyte proteins (85%; 11/13) were decreased. In AD CAA(+) vessels, there was enrichment for perivascular fibroblast (*p *= 1.26 × 10^–2^) and oligodendrocyte (*p *= 5.80 × 10^–3^) proteins. The majority of perivascular fibroblast proteins (60%; 3/5) were decreased and half of the annotated oligodendrocyte proteins (50%; 7/14) were decreased.

Top significant GOCC terms (FDR adjusted *p* value < 0.05) were determined for the differentially abundant proteins (Fig. 4c and d, Supplementary Tables 4–5). The most significant GOCC term associated with increased proteins for both MCI and AD was collagen-containing extracellular matrix (MCI: adj. *p *= 4.15 × 10^–14^, 26 proteins; AD: adj. *p *= 1.51 × 10^–6^, 29 proteins). The most significant GOCC term associated with decreased proteins for both MCI and AD was ribonucleoprotein complex (MCI: adj. *p *= 1.12 × 10^–8^, 44 proteins; AD: adj. *p *= 3.33 × 10^–19^, 53 proteins).

Collagen protein subtypes were notably increased in CAA(+) vessels in both MCI and AD. In MCI, increased collagen proteins included 8 proteins from the collagen I, IV, VI, and XVIII families, and for AD 2 proteins from the IV and XVIII families (Supplementary Table 3, Supplementary Fig. 1). In addition to the altered collagen proteins associated with the GOCC collagen-containing extracellular matrix term shared by both MCI and AD, this GOCC term also included increased laminin proteins (LAMA5, LAMC1), APOE, CLU, SMOC1, TGFBI, HTRA1, EFEMP1, NPNT, SPON1, and EMILIN1.

Ribosome proteins and ribonucleoproteins were notably decreased in both MCI and AD CAA(+) vessels. Of the proteins associated with the GOCC ribonucleoprotein complex term, 22 proteins were shared between both disease groups (unique proteins: MCI 22, AD 31). Key BBB proteins that had robust expression in CAA(−) vessels were significantly decreased in CAA(+) vessels (Supplementary Fig. 2), including OCLN (MCI: 2.7-fold decrease, *p *= 2.03 × 10^–2^; AD: not detected), TJP1 (also known as zona occludens-1; ZO-1; MCI: 2.7-fold decrease, *p *= 4.45 × 10^–3^; AD: 1.7-fold decrease, *p *= 1.76 × 10^–3^), and SLC2A1 (also known as GLUT1; MCI: 2.2-fold, *p *= 0.059; AD: 1.7-fold, *p *= 0.030).

**Fig. 5:**
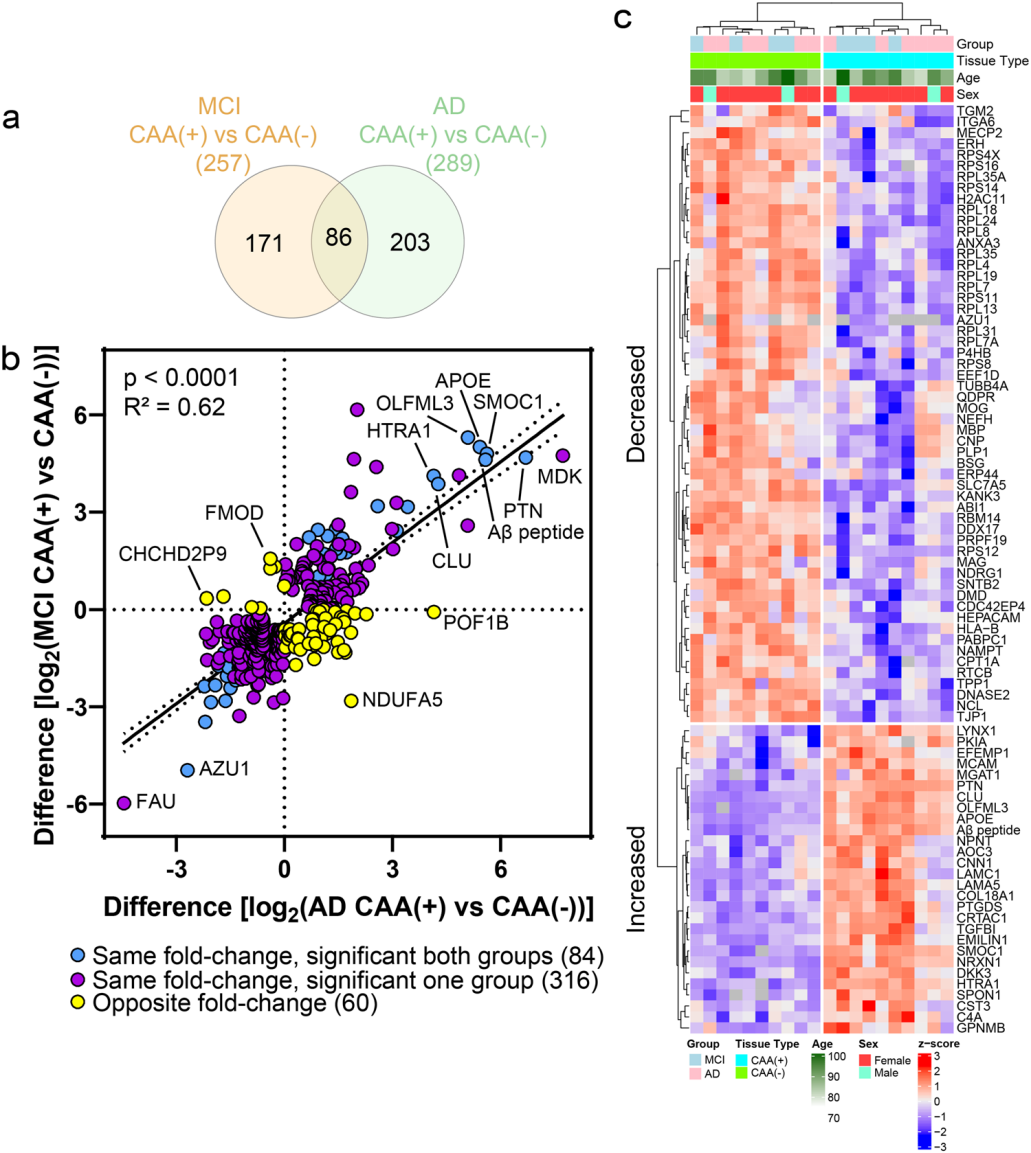
CAA(+) vs CAA(−) vessel protein differences positively correlate in MCI and AD. **a** Of the differentially abundant proteins in CAA(+) vessels when compared to CAA(−) vessels, 86 were shared in MCI and AD. Of the 86, 84 changed in the same fold-change direction. **b** Of differentially abundant proteins in at least one disease group (460 proteins), there was a positive correlation of protein changes (*p *< 0.0001, *R*^2^ = 0.62). There were 87% (400/460) of proteins changing in the same direction (purple) and 13.0% (60/460) proteins changing in the opposite direction (yellow). The 84 proteins changing in the same fold-change direction and significant in both disease groups are indicated in blue. Proteins of interest are annotated by gene name. **c** The 84 shared significant CAA(+) vessel proteins in MCI and AD were evaluated by unsupervised hierarchical clustering, and indicated clustering by tissue type [CAA(+) or CAA(−)]. Expression levels are depicted by z-score in the heatmap. The top cluster are proteins decreased in CAA(+) vessels and the bottom cluster are the proteins increased in CAA(+) vessels

Protein changes in CAA(+) vessels were similar in MCI and AD. There were 86 proteins significantly altered in both MCI and AD, of which 84/86 were changed in the same fold-change direction (Fig. 5a). The remaining two proteins (DNA2 and WIPF3) were significantly decreased in CAA(+) vessels in MCI and increased in AD. While there were seemingly many proteins uniquely altered in only one group (Fig. 5a), there was a strong positive correlation (*p *< 0.0001, *R*^2^ = 0.62) between protein changes in MCI and AD (Fig. 5b) suggesting that differences between groups were likely due to the power of our study rather than biologic differences. There were 87% (400/460) of proteins altered in either MCI or AD that changed in the same fold-change direction (Fig. 5b). The 84 shared significant CAA(+) vessel proteins were evaluated by unsupervised hierarchical clustering, which indicated clustering by tissue type (Fig. 5c). Of those proteins altered in both disease groups, there was particular representation of decreased ribosome proteins, as was also seen most significantly by GOCC enrichment.

### Non-CAA vessel comparisons

**Fig. 6:**
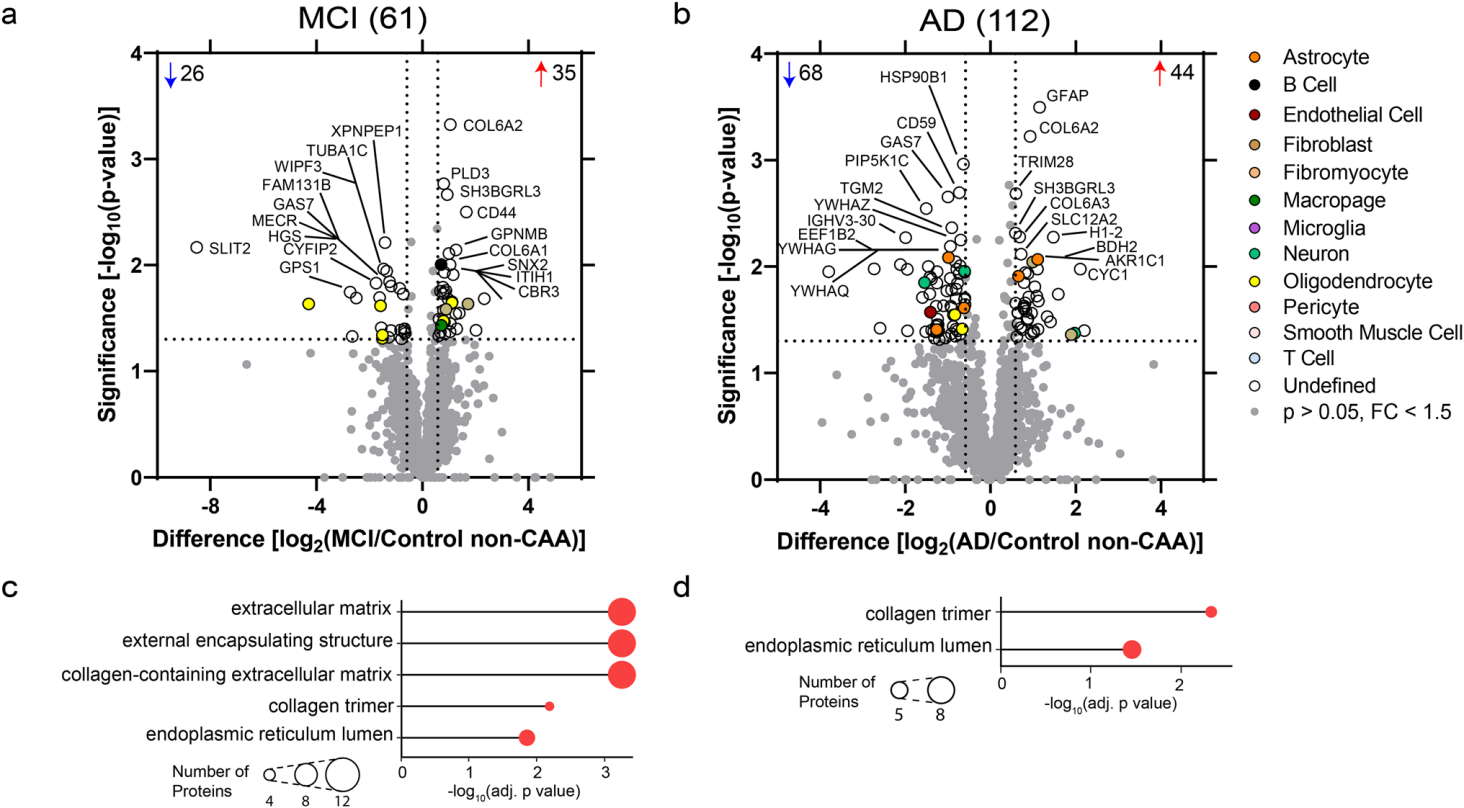
Non-CAA vessel differential expression analysis and GO cell component terms in MCI and AD. **a** In MCI CAA(−) vessels when compared to Control CAA(−) vessels, there were 61 proteins differentially abundant (*p *< 0.05, fold change > 1.5), with 35 increased (red) and 26 decreased (blue). The top ten increased and decreased are annotated by gene name. **b** In AD CAA(−) vessels when compared to Control CAA(−) vessels, there were 112 proteins differentially abundant, with 44 increased and 68 decreased. **c** and **d** Differentially abundant proteins were associated with the indicated increased (red) GOCC terms at adj. *p *< 0.05 in MCI and AD. Number of proteins associated with a term are depicted by circle size

Significant protein changes were also observed in CAA(−) vessels in MCI and AD. To identify protein differences in CAA(−) vessels from MCI or AD cases, CAA(−) vessels in both disease groups were compared to Control CAA(−) vessels by unpaired *t* tests at *p *< 0.05 and with fold change > 1.5. There were 61 proteins differentially abundant in MCI, and 112 proteins in AD (Fig. 6a and b).

After cell-type annotation analysis, the majority of proteins were “Undefined” as they are expressed by multiple cell types or it is unknown. In MCI CAA(−) vessels, there was enrichment for perivascular fibroblast (*p *= 4.12 × 10^–2^) and oligodendrocyte (*p *= 1.90 × 10^–3^) proteins. Both of the perivascular fibroblast proteins (100%; 2/2) were increased and the majority of the oligodendrocyte proteins (67%; 4/6) were decreased. In AD CAA(−) vessels, there was enrichment for neuron (*p *= 4.30 × 10^–3^) proteins, with the majority decreased (75%; 3/4).

Top significant GOCC terms (FDR adj. *p *< 0.05) were determined for the differentially abundant proteins (Fig. 6c and d, Supplementary Tables 6–7). The most significant GOCC terms associated with increased proteins for MCI included three terms: extracellular matrix, external encapsulating structure, and collagen-containing extracellular matrix (adj. *p *= 5.57 × 10^–4^, 12 proteins for all three terms). For AD, the top significant GOCC term was collagen trimer (adj. *p *= 4.65 × 10^–3^, 5 proteins). Decreased proteins were not significantly associated with GOCC terms (Supplementary Tables 6–7).

Among the most significantly increased proteins, multiple collagen protein subtypes were increased in both MCI and AD CAA(−) vessels. In MCI, increased collagen proteins included 4 proteins from the VI and XII families, and for AD 5 proteins from I, VI, and XII families (Supplementary Table 3, Supplementary Fig. 1).

**Fig. 7:**
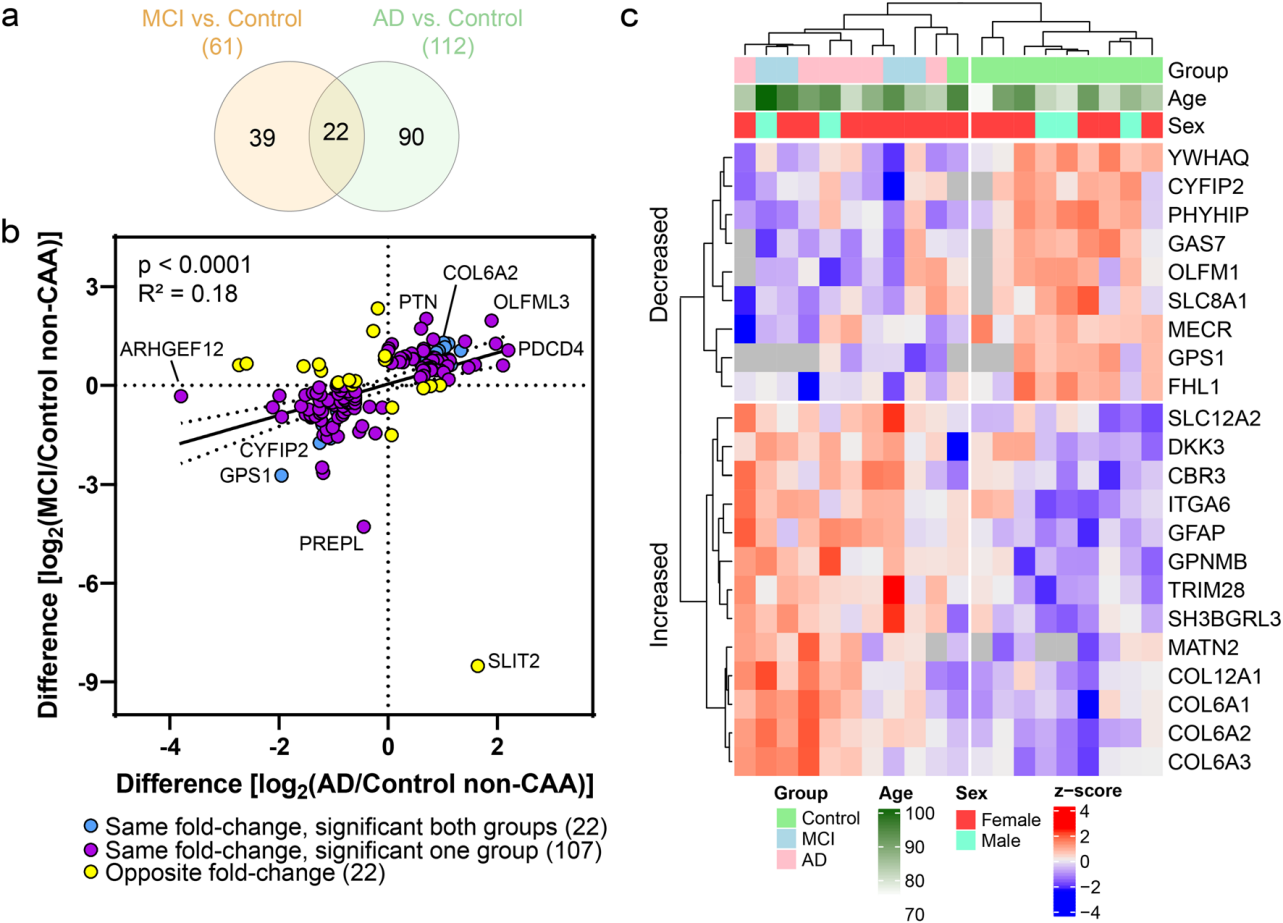
Non-CAA vessel protein differences have a mild positive correlation in MCI and AD. **a** Of the differentially abundant proteins in MCI or AD CAA(−) vessels when compared to Control CAA(−) vessels, 22 were shared and all changed in the same fold-change direction. **b** Of differentially abundant proteins in at least one disease group (151 proteins), there was a mild positive correlation of protein changes (*p *< 0.0001, *R*^2^ = 0.18). There were 85% (129/151) of proteins changing in the same direction (purple) and 14.6% (22/151) proteins changing in the opposite direction (yellow). The 22 proteins changing in the same fold-change direction and significant in both disease groups are indicated in blue. Proteins of interest are annotated by gene name. **c** The 22 shared significant CAA(−) vessel proteins in MCI and AD were evaluated by unsupervised hierarchical clustering, which indicated clustering of Control cases from MCI and AD cases. Expression levels are depicted by z-score in the heatmap. The top cluster are proteins decreased in MCI and AD CAA(−) vessels, and the bottom cluster are the proteins increased in MCI and AD CAA(−) vessels

**Fig. 8:**
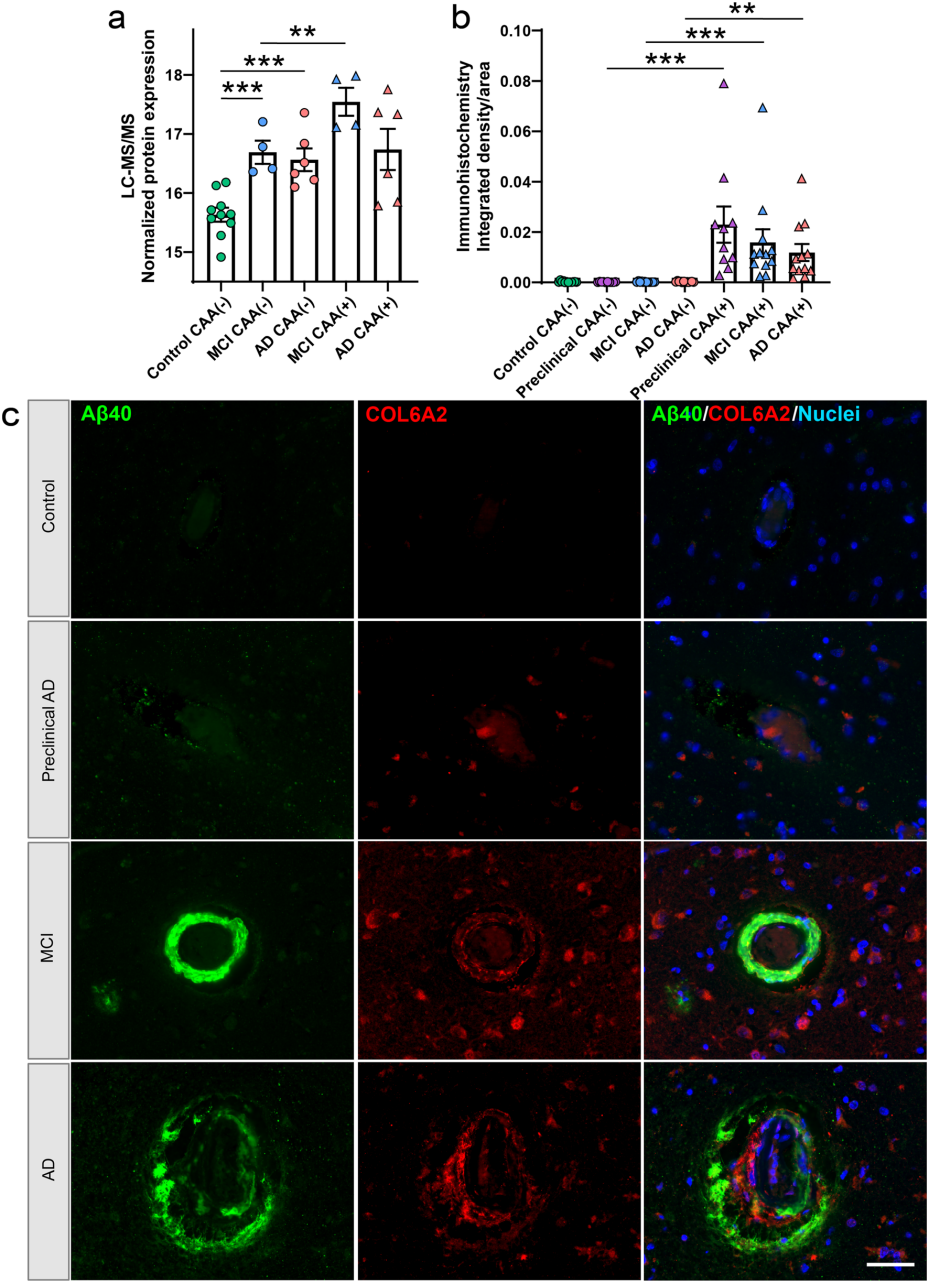
COL6A2 histologic characterization. **a** Proteomics quantification of COL6A2 showed enrichment in CAA(−) vessels in both MCI and AD, as well as in CAA(+) vessels from MCI. **b** Quantification of COL6A2 immunoreactive area in CAA(+) and CAA(−) vessels in temporal cortex sections from progressive stages of disease, Control (*n *= 12), Preclinical AD (*n *= 10), MCI (*n *= 12), and AD (*n *= 12), showed enrichment in preclinical AD, MCI, and AD CAA(+) vessels. **c** Representative images show COL6A2 immunoreactivity in CAA at progressive stages of disease. Significant pairwise comparisons are indicated for those analyses that were performed, ** *p *< 0.01, *** *p *< 0.0001. Scale bar = 50 µm

Of the differentially abundant proteins, 22 were shared by both MCI and AD and all changed in the same fold-change direction (Fig. 7a). Among significant proteins in at least one disease group (151 proteins), there was a mild positive correlation (*p *< 0.0001, *R*^2^ = 0.18) with 85% (129/151) changing in the same fold-change direction (Fig. 7b). The 22 shared significant CAA(−) vessel proteins were evaluated by unsupervised hierarchical clustering, which indicated clustering of Control from MCI and AD CAA(−) vessel samples (Fig. 7c). Shared proteins included several increased collagens (COL6A1, COL6A2, COL6A3, COL12A1). As COL6A2 was previously associated with CAA severity [20], we selected COL6A2 for further validation using immunohistochemistry in the current study. We observed increased intensity of COL6A2 in CAA(+) vessels of preclinical AD, MCI, and AD cases and not in CAA(−) vessels (Fig. 8). Among those proteins significant in only one disease group, MCI included increased ribosome proteins (RPL28, RPL35A, RPS10), and AD included decreased 14-3-3 family proteins (YWHAB, YWHAE, YWHAG, YWHAH, YWHAZ; as well as YWHAQ in both AD and MCI).

### Comparison of CAA and plaque proteomic studies

**Fig. 9:**
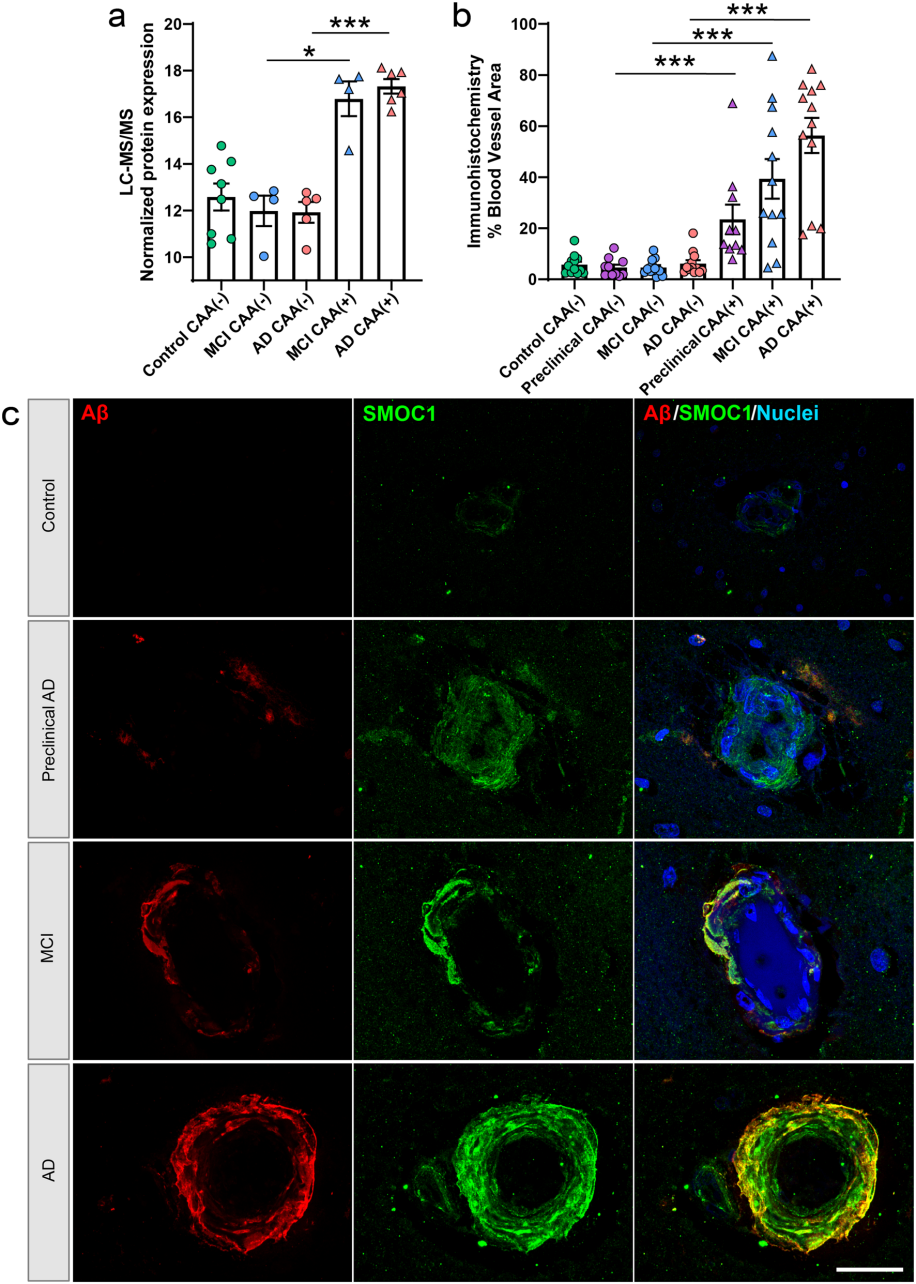
SMOC1 histologic characterization. **a** Proteomics quantification of SMOC1 showed enrichment in CAA(+) vessels in both MCI and AD. **b** Quantification of SMOC1 immunoreactive area in CAA(+) and CAA(−) vessels in temporal cortex sections from progressive stages of disease, control (*n *= 12), preclinical AD (*n *= 10), MCI (*n *= 12), and AD (*n *= 12), showed enrichment in preclinical AD, MCI, and AD CAA(+) vessels, similar to proteomic quantification. **c** Representative images show SMOC1 immunoreactivity in CAA at progressive stages of disease. Significant pairwise comparisons are indicated for those analyses that were performed, *p < 0.05, ***p < 0.0001. Scale bar = 25 µm

**Fig. 10:**
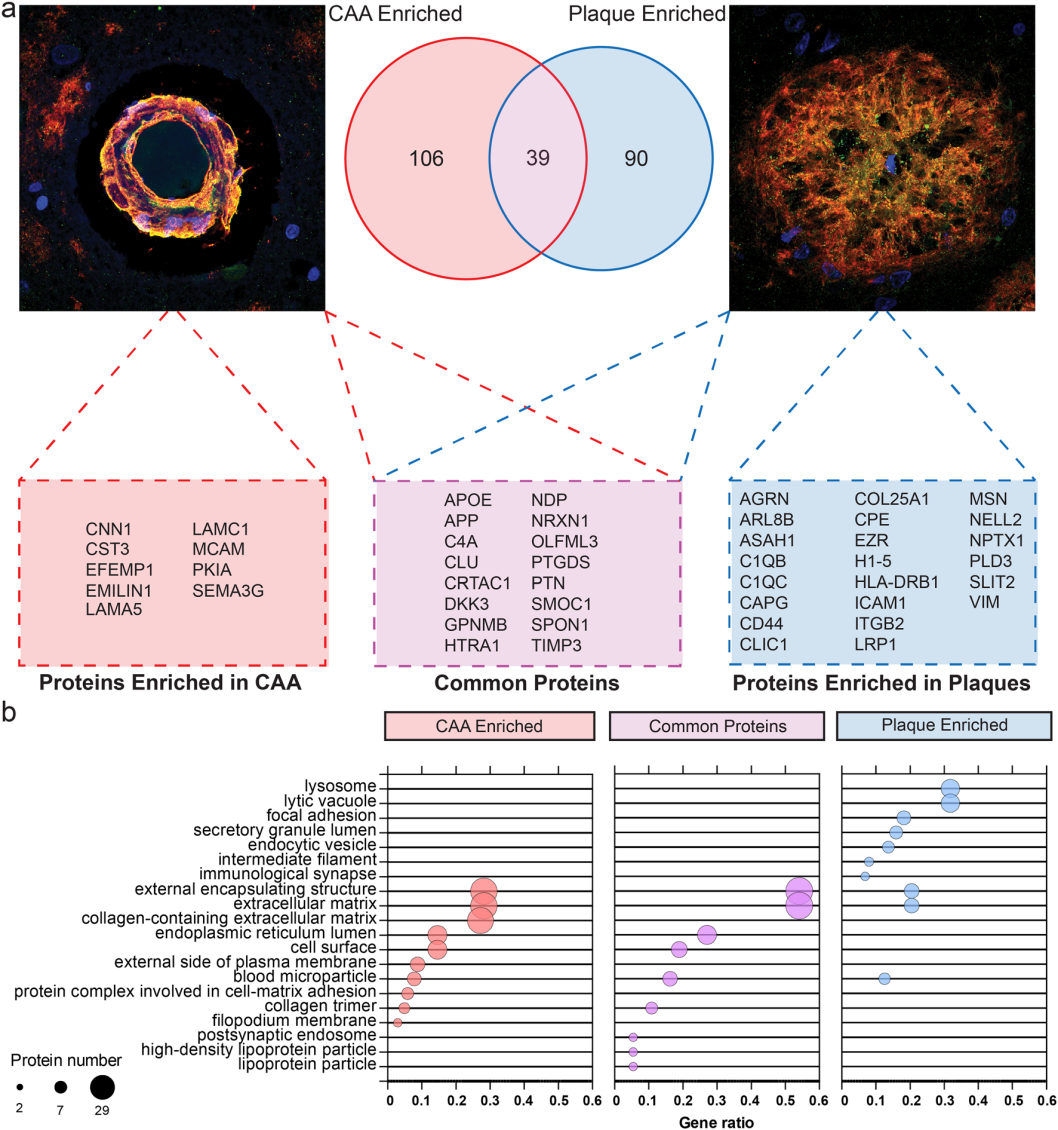
Potential CAA and Aβ plaque protein markers. **a** Schema highlighting key proteins detected as enriched in CAA(+) vessels compared to plaque-enriched proteins from Drummond [10] and Xiong [73], which may act as markers for these pathologies. Venn diagram shows the overlap between the two pathologies. Boxes show examples of high confidence proteins in each group. Representative images of CAA and plaques: blue = nuclei (Hoescht), green = SMOC1 (example extracellular matrix protein), and red = Aβ. **b** Top 10 GOCC terms for each subgroup (potential CAA marker, potential plaque marker, and common proteins). GOCC terms have been simplified to reduce similar terms. Red = CAA enriched, blue = plaque enriched, and magenta = common proteins

**Fig. 11:**
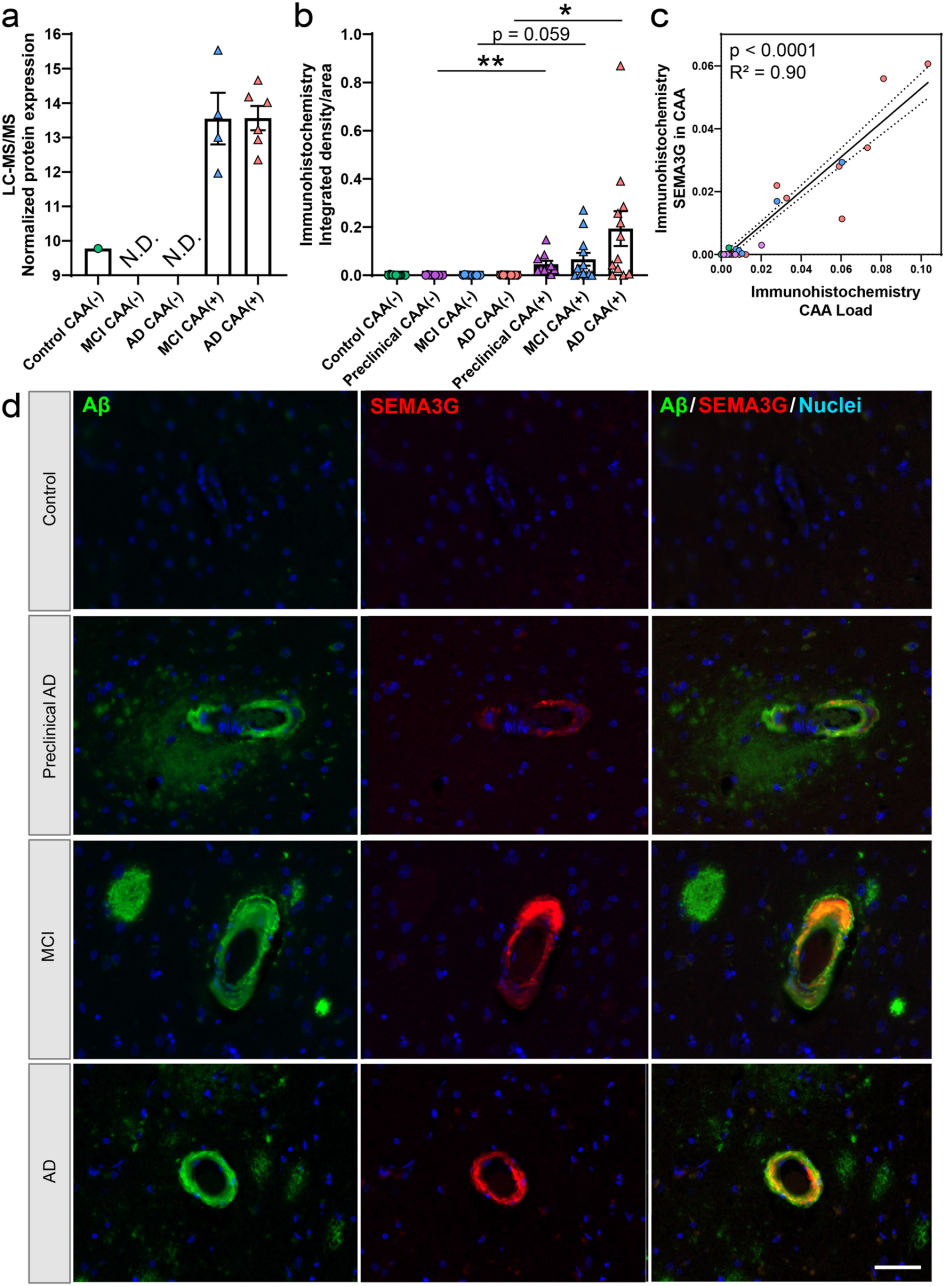
SEMA3G histologic characterization. **a** Proteomics quantification of SEMA3G showed enrichment in CAA(+) vessels in both MCI and AD. *N.D.* not detected. **b** Quantification of SEMA3G immunoreactive area in CAA(+) and CAA(−) vessels in temporal cortex sections from progressive stages of disease, Control (*n *= 12), Preclinical AD (*n *= 10), MCI (*n *= 12), and AD (*n *= 12), showed enrichment in preclinical AD, trend in MCI, and enrichment in AD CAA(+) vessels. **c** Histologic quantification of SEMA3G in CAA(+) vessels correlated to CAA levels (*p *< 0.0001; *R*^2^ = 0.90). **d** Representative images show SEMA3G immunoreactivity in CAA at progressive stages of disease. Significant pairwise comparisons are indicated for those analyses that were performed, *p < 0.05, **p < 0.01. Scale bar = 50 µm

Amyloid-associated proteins were evaluated further to identify CAA(+) vessel and amyloid-plaque-enriched proteins. The CAA proteomic results in this study were compared to two datasets that identified proteins significantly enriched or depleted in amyloid plaques relative to neighboring non-plaque tissue from sporadic AD, preclinical AD, early onset AD, and Down syndrome cases [10, 73]. This comparison showed that for proteins identified in both CAA and plaque datasets, 39/235 CAA enriched proteins were also enriched in amyloid plaques. Notably, this included 9 proteins that were significantly enriched in both CAA and plaques in all experimental groups examined in all 3 datasets (APP, APOE, SMOC1, SPON1, GPNMB, C4A, HTRA1, OLFML3, NRXN1), indicating a particularly robust enrichment of these proteins in both CAA and plaques. We validated the enrichment of one of these proteins, SMOC1, in CAA using immunohistochemistry and confirmed significant enrichment of SMOC1 in CAA(+) vessels in preclinical AD, MCI, and advanced AD (Fig. 9). There were also 106 proteins that were potentially only enriched in CAA and 90 proteins that were potentially only enriched in plaques (Fig. 10, Supplementary Table 8). Of these, SEMA3G emerged as a promising potential CAA specific marker, as it was detected in CAA(+) vessels from both MCI and AD, was not detected or at low levels in CAA(−) vessels (detected in one control), correlated to Aβ peptide levels (*p *= 0.0016; *R*^2^ = 0.69), and was not detected in the previous plaque proteomic studies. SEMA3G was evaluated by immunohistochemistry and showed enrichment in CAA(+) vessels in preclinical AD, trending in MCI, and enrichment in advanced AD (Fig. 11), with variation in CAA levels. Immunohistochemical SEMA3G levels correlated with CAA pathology levels (*p *< 0.0001; *R*^2^ = 0.90). While the other proteins reported to be enriched in either CAA or plaques could indicate proteins that selectively accumulate in one type of pathology, these are preliminary findings only, given the likely presence of false negative results based on the low sample sizes used to generate currently available amyloid-plaque-enriched protein datasets. Comparison of functional associations showed that proteins enriched in both CAA and plaques, proteins enriched in CAA, and proteins enriched in plaques largely belonged to the same protein networks, particularly collagen-containing extracellular matrix proteins (Fig. 10b) that suggest proteins enriched in CAA and plaques are largely similar.

## Discussion

This is the first study to evaluate both the CAA(+) and neighboring CAA(−) vessel proteomes separately, as well as first to evaluate the vessel proteomes in MCI. We identified 257 proteins that were differentially abundant in CAA(+) vessels in MCI and 289 proteins that were differentially abundant in CAA(+) vessels in AD, most significantly associated with increased collagen-containing extracellular matrix and decreased ribonucleoprotein complex and BBB proteins. We found that the CAA(+) vessel proteome was similar in MCI and AD cases. Interestingly, we also observed differences in CAA(−) vessels in both MCI and AD in comparison to Control cases, suggesting changes in blood vessel integrity in the absence of CAA. Finally, a comparison between the CAA and amyloid-plaque proteomes confirmed that many of the same amyloid-associated proteins were enriched in both CAA and plaques.

In CAA(+) vessels, many collagen proteins were increased in both MCI and AD. Collagens are a component of the basement membrane, which undergoes thickening with aging and in AD [65]. Similar to previous histologic studies [15], we identified increased collagen IV proteins, as well as collagen I, VI, and XVIII family proteins for MCI and XVIII family proteins in AD. These findings extend a previous CAA proteomics study that reported elevated COL6A2 in CAA cases [20], as we validated here. Further, basement membrane protein changes have been observed in previous studies that in addition to increased collagen IV included fibronectin, agrin, and perlecan (also known as HSPG2) [24]. Similarly, we identified increased collagen IV subtype proteins in CAA(+) vessels for COL4A1, COL4A2 in MCI and decreased COL4A3 in AD; with trends seen across disease groups. By contrast, we did not see changes in fibronectin or agrin in the pairwise comparisons evaluated. Increased collagens in vessels with Aβ deposition suggest vascular matrix reorganization, which may contribute to vascular dysfunction in both MCI and AD.

Proteins enriched in CAA and plaques were largely similar, both at the individual protein level and at the protein network level. Proteins enriched in both CAA and plaques included many present in the matrisome protein cluster previously reported to strongly correlate with amyloid pathology, tau pathology and cognitive status (i.e., APP, APOE, SMOC1, SPON1, GPNMB, HTRA1, OLFML3, PTN, SPOCK2, NTN1, MD) [27, 37]. Our results provide new evidence that many of these matrisome module proteins are highly enriched in CAA in human AD brain tissue and further support the hypothesis that accumulation of extracellular matrix proteins is a key feature of both CAA and plaque pathology. While many proteins had apparent enrichment in either CAA or plaques, these results should be interpreted cautiously as they are reliant on low-powered datasets. While we provide some preliminary findings about proteins that may be enriched in either CAA or plaques, knowledge in this area will continue to evolve as future higher-powered studies examining CAA or plaque-enriched proteins are published. One protein that did emerge as different in plaques and CAA was semaphorin 3G (SEMA3G), which is a class 3 secreted semaphorin [63]. Our results showed enrichment of SEMA3G in CAA(+) vessels, correlation to regional CAA levels, which was not detected in other CAA proteomic studies with the approaches used [20, 23, 26, 42, 45, 72, 75], not detected in plaque tissue [10, 11], and not detected in other AD proteomic studies [1]. Further, a recent study indicated that SEMA3G secretion in the brain modulates synaptic function in an animal model [62]. SEMA3G is detectable in human CSF [62, 69, 72], making it a potential diagnostic biomarker of CAA. These results indicate that SEMA3G may potentially function as a biomarker of CAA. There are currently no biomarkers for CAA, thus this may facilitate CAA diagnosis and evaluation of patients at risk for ARIA when administered currently available immunotherapies.

Many proteins were significantly decreased in CAA(+) vessels, most significantly ribonucleoprotein complex and ribosome proteins in both MCI and AD, which are associated with protein translation. Previous mouse models show decreased protein synthesis occurs early with aging [59], and AD mouse model studies indicate decreased ribosomal proteins correlate to increased phosphorylated tau (pTau) levels [14]. Further, ribosome protein expression is linked to the cell stress response [29, 49]. Previous CAA proteomics studies have not identified ribonucleoprotein complex protein differences with the approaches used [20, 23, 26, 72, 75], although some protein differences are seen with other pairwise comparisons when evaluating aging and low CAA to age-matched Control cases. Other non-CAA studies have linked the ribonucleoprotein HNRNPA1 to APP splicing, with a suggestion that increased HNRNPA1 levels may be protective [39]. HNRNPA1 was decreased in MCI CAA(+) vessels and a similar trend was observed in AD CAA(+) vessels, as well as CAA(−) vessels, thus HNRNPA1 may be associated with development and/or progression of vessel pathology.

Of particular interest among decreased proteins in CAA(+) vessels were altered proteins important for BBB integrity, including tight junction proteins, SLC2A1 (GLUT1), and basement membrane proteins. OCLN and TJP1 were decreased in CAA(+) vessels of both MCI and AD cases, which was not different or not detected in other human CAA proteomics studies with the approaches used [20, 23, 72, 75]. In previous non-proteomic CAA studies, TJP1 was decreased histologically in frontal cortex, TJP1 and OCLN were decreased in leptomeninges as measured biochemically, and OCLN and TJP1 were decreased in occipital lobe histologically in CAA cases with capillary involvement [5, 7, 17]. In AD, with cases having a range of CAA severity, changes to BBB proteins included decreased OCLN in several cortical regions as well as correlations to CAA severity particularly in the inferior temporal cortex as measured by ELISA in brain homogenate relative to CD31 levels [74]. Changes in TJP1 and OCLN have also been reported in AD animal models [17, 58]. SLC2A1 (GLUT1) is expressed by endothelial cells [56], and it is enriched in vessels as measured by proteomics but not different by CAA status in mixed Aβ+/− vessels [20]. When SLC2A1 is decreased it is associated with vascular dysfunction [15], and it is decreased as measured by other approaches in AD as well as indirectly in MCI on brain imaging [15, 76]. Similarly in the current study, SLC2A1 was detected in all samples, with a decrease in AD and trending decrease in MCI CAA(+) vessels. In addition to the collagen basement membrane proteins, metalloproteinases (MMPs) and their inhibitors (TIMPs) [8] also contribute to BBB integrity. Previous histologic studies in occipital lobe showed an altered ratio of MMP9 and TIMP3 in leptomeningeal vessels of CAA cases with intracerebral hemorrhage [28]. MMP proteins were not detected in the current study, but TIMP3 was detected in all CAA(+) vessel samples as well as one Control CAA(−) vessel sample indicating that the inhibitor of MMP9 is elevated in CAA(+) samples. Our results support deficits in BBB integrity and vascular function in both MCI and AD, which are relevant to the underlying disease mechanisms and provide implications for therapeutics like parenchymal drug delivery and ARIA-related risk associated with recently available immunotherapies [53, 60].

In neighboring CAA(−) vessels, there were protein changes identified in both MCI and AD that were associated with increased collagens and the extracellular matrix. In MCI, increased collagen proteins included 4 proteins from the VI and XII families, and for AD 5 proteins from I, VI, and XII families. Additionally, proteins altered only in AD CAA(−) vessels included decreased 14-3-3 family proteins, with similar trends seen in MCI CAA(−) vessels as well as in CAA(+) vessels of both MCI and AD. From previous CAA proteomics studies, this protein family was not altered [20, 23, 75], and was decreased in brain tissue from some AD proteomic studies and increased or present in plaque proteomic studies [1]. The 14-3-3 proteins are phospho-binding proteins that have many cellular regulatory functions, and in AD colocalize with neurofibrillary tangles, interact with AD pathology associated proteins, and are decreased in AD choroid plexus when compared to Control cases [16, 33, 46]. Proteomic differences in CAA(−) vessels from those cases with CAA is expected, as the mechanisms of CAA previously described indicate Aβ deposition occurs in a spiral-like patchy pattern [6] and thus it would be expected that neighboring cells without Aβ deposition are impacted. Further, it is proposed that there is a bidirectional relationship between vascular dysfunction and Aβ clearance [15]. To expand on this point, our results in CAA(−) vessels may include a mix of protein changes as a result of both the effects of reactive changes from neighboring Aβ accumulation as well as protein changes that may occur before Aβ deposition as a consequence of various genetic and/or environmental factors (diet, exercise, stress) [43]. To better understand these protein changes, it will be of interest in future studies to evaluate protein differences that correspond to disease duration, CAA pathology severity, differences across brain regions (i.e., temporal lobe vs. the more CAA vulnerable occipital lobe), and the contribution of other co-occurring vascular pathologies to protein changes, i.e., hypertension, infarction, atherosclerosis.

There were several limitations to our study, including small sample size that was hindered by the low levels of CAA in the inferior temporal cortex. Our technique is less sensitive in detecting membrane proteins, insoluble proteins, and low abundance proteins. Heterogeneous clinical variables warrant further evaluation in future studies with larger samples, including age, disease duration, *APOE* genotype, neuropathology across the severity spectrum, cognitive scores, medications, co-occurring vascular pathologies.

In summary, we identified protein changes in CAA(+) and neighboring CAA(−) vessels in both MCI and AD that were associated with vascular matrix reorganization, protein translation deficits, and BBB breakdown. These changes can contribute to vascular dysfunction and inform future mechanistic, therapeutic, and biomarker studies.

### Supplementary Information

Below is the link to the electronic supplementary material.

Supplementary file1 (DOCX 387 kb)

Supplementary file2 (XLSX 2022 kb)

## Data Availability

Data are available in the supplementary data files. In addition, the mass spectrometric raw files are accessible at https://massive.ucsd.edu under accession MassIVE MSV000094400.
