# Supplementary material for: Differences in the cerebral amyloid angiopathy proteome in Alzheimer’s disease and mild cognitive impairment
Source: Acta Neuropathol. 2024 Jul 22;148(1):9. doi: 10.1007/s00401-024-02767-1 (PMC11263258; doi:10.1007/s00401-024-02767-1)
Supplement: Supplementary file 1 — Supplementary file1 (DOCX 387 kb) [file 401_2024_2767_MOESM1_ESM.docx]

**Differences in the Cerebral Amyloid Angiopathy Proteome in Alzheimer’s Disease and Mild Cognitive Impairment**

Dominique Leitner^1,2,3^, Tomas Kavanagh^4^, Evgeny Kanshin^5,6^, Kaleah Balcomb^4^, Geoffrey Pires^1,3^, Manon Thierry^1,3^, Jianina I. Suazo^1,3^, Julie Schneider^7,8.9^, Beatrix Ueberheide^5,6^, Eleanor Drummond^4*^, Thomas Wisniewski^1,3,10,11*^

**Supplemental Materials**

**Supplemental Figure 1. Collagen proteins. a-m)** All detected collagen proteins are depicted. Significant pairwise comparisons are indicated for those analyses that were performed, * p < 0.05, ** p < 0.01, *** p < 0.0001.

**Supplemental Figure 2. Proteins important for blood brain barrier function. a)** Occludin (OCLN) and **b)** TJP1 (ZO-1) were decreased or not detected (N.D.) in CAA(+) vessels when compared to CAA(-) vessels. **c)** SLC2A1 (GLUT1), important to the function of the BBB, was decreased in AD CAA(+) vessels, with a similar trend seen in MCI. Significant pairwise comparisons are indicated for those analyses that were performed, * p < 0.05, ** p < 0.01, *** p < 0.0001.

**Supplemental Table 1. Detailed Case History**

**Supplemental Table 2. Multiple Linear Regression of PCA for Group and Tissue Type**

**Supplemental Table 3. LFQ Normalized Data, CAA(+) vs CAA(-) Paired T-tests, CAA(-) Unpaired T-tests**

**Supplemental Table 4. GO Cell Component Terms for MCI CAA(+) vs CAA(-)**

**Supplemental Table 5. GO Cell Component Terms for AD CAA(+) vs CAA(-)**

**Supplemental Table 6. GO Cell Component Terms for MCI vs. Control non-CAA**

**Supplemental Table 7. GO Cell Component Terms for AD vs. Control non-CAA**

**Supplemental Table 8. CAA and Amyloid Plaque Proteome Comparison**

**Supplemental Figure 1.**

**
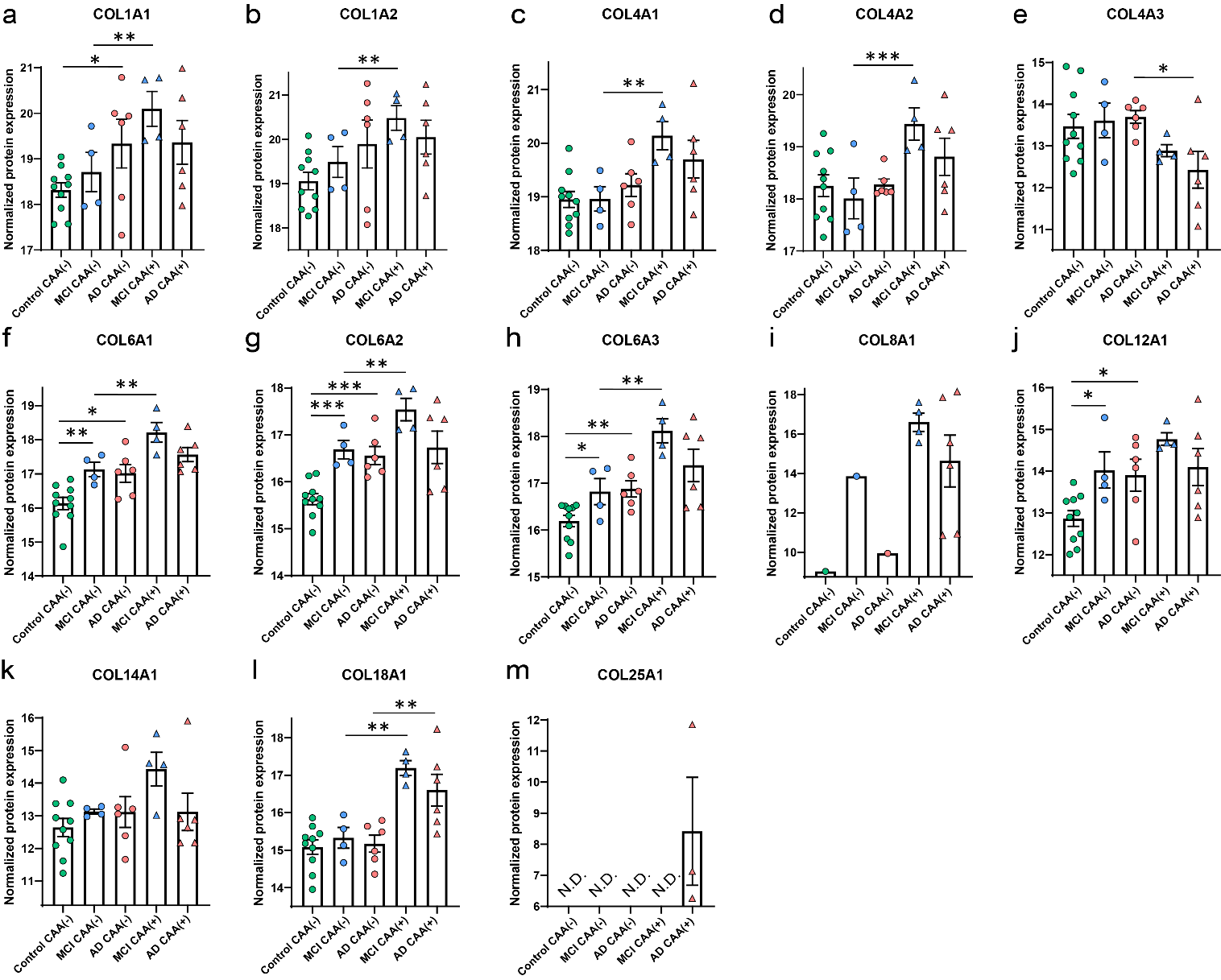
**

**Supplemental Figure 2.**

**
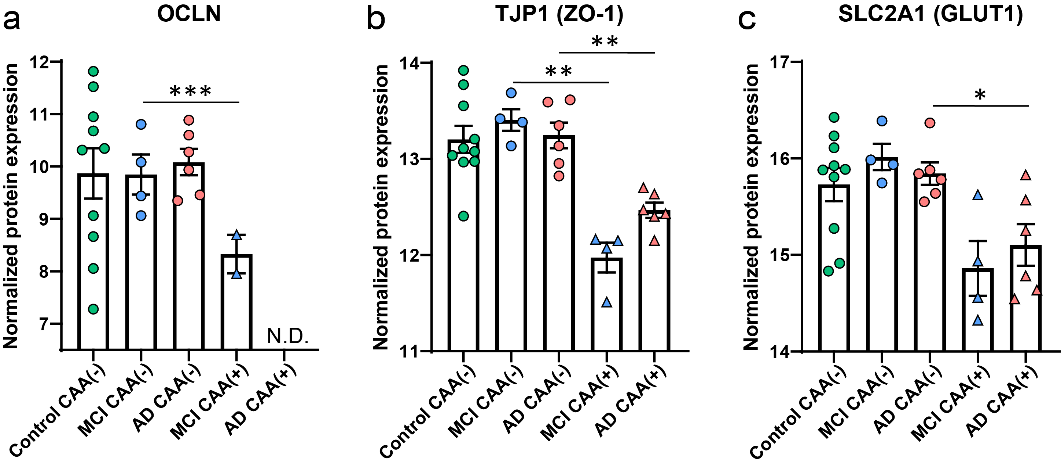
**
